# Supplementary material for: Clonal lineage tracing of innate immune cells in human cancer
Source: bioRxiv. 2025 Jul 21:2025.07.16.665245. Preprint. [Version 1] doi: 10.1101/2025.07.16.665245 (PMC12330704; doi:10.1101/2025.07.16.665245)
Supplement: Supplement 2 [file NIHPP2025.07.16.665245v1-supplement-2.pdf]

bioRxiv preprint doi: https://doi.org/10.1101/2025.07.16.665245; this version posted July 21, 2025. The copyright holder for this preprint (which was not certified by peer review) is the author/funder, who has granted bioRxiv a license to display the preprint in perpetuity. It is made available under aCC-BY 4.0 International license.

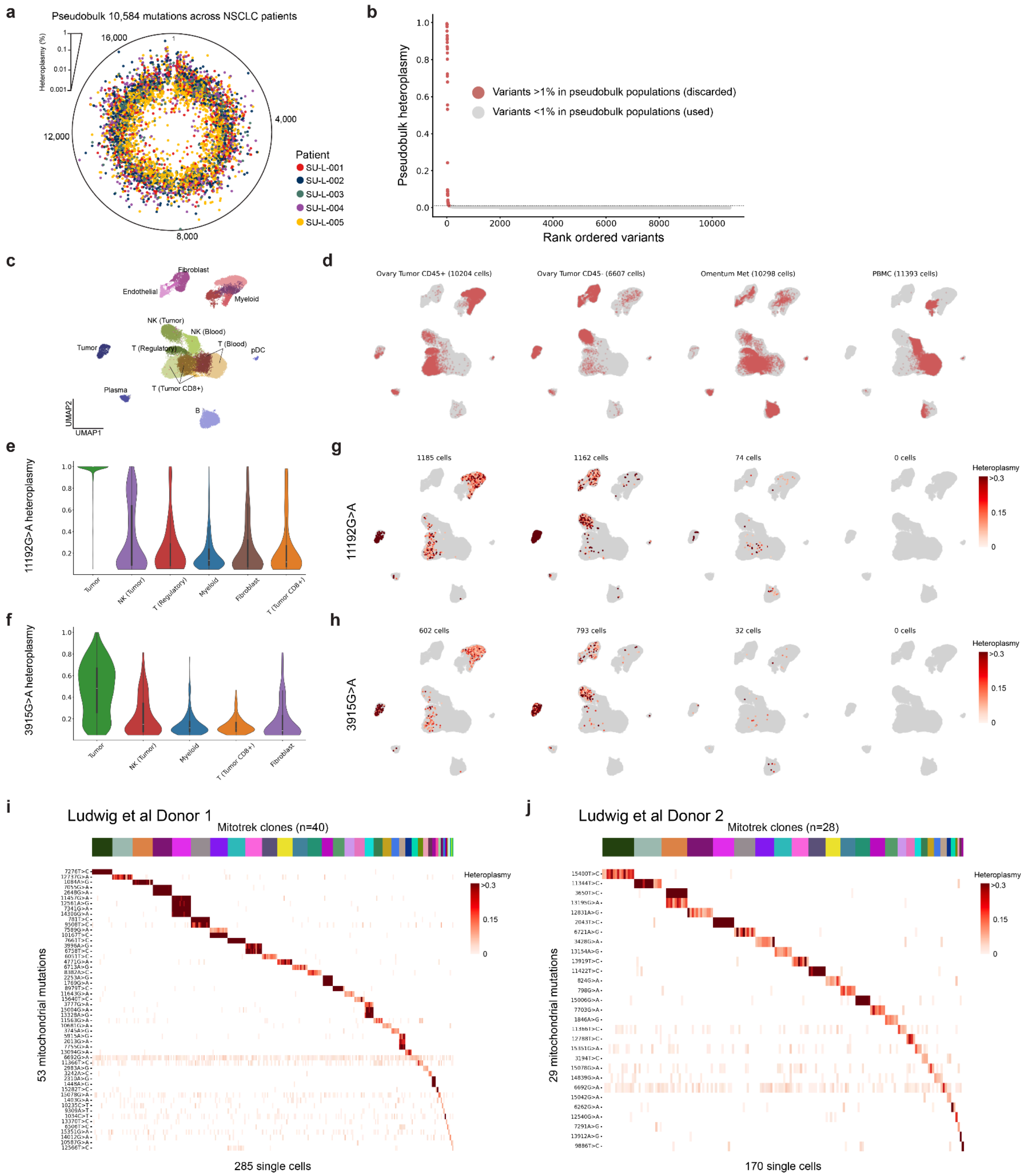

Supplemental Figure 1: Benchmarking Mitotrek using ground-truth data

(A) Distribution of mgatk-nominated variants along the mitochondrial genome, averaged across cells and colored by patient.

bioRxiv preprint doi: https://doi.org/10.1101/2025.07.16.665245; this version posted July 21, 2025. The copyright holder for this preprint (which was not certified by peer review) is the author/funder, who has granted bioRxiv a license to display the preprint in perpetuity. It is made available under aCC-BY 4.0 International license.

(B) Pseuboulk heteroplasmy for all variants detected by mgatk in across all samples. Variants with >1% pseudobulk heteroplasmy are excluded from downstream analysis. 1% is chosen as the conservative threshold after observing the overall heteroplasmy distribution.

(C) UMAP embeddings of tumor-infiltrating immune cells from matched primary (ovarian) and metastatic (omentum) tumors, and PBMCs from HGSC patient SU-O-005.

(D) Distribution on the UMAP of cells from indicated samples. Cells from the primary ovarian tumor were sorted by CD45 to separate tumor-infiltrating immune cells.

(E and F) Heteroplasmy levels of the indicated tumor-specific mitochondrial variants in tumor-infiltrating immune cells processed together with tumor cells.

(G and H) Mutations projected onto UMAP embeddings across samples. Tumor-specific variants are indiscriminately detected at lower heteroplasmy levels in all cells from the same sample, suggesting mitochondrial transfer and/or technical artifacts (ambient mtDNA).

(I and J) Heatmap showing the heteroplasmy levels of variants (rows) that are identified as clone markers to group cells (columns) in each Mitotrek clone. Position of each variant and the base pair change are shown.

bioRxiv preprint doi: https://doi.org/10.1101/2025.07.16.665245; this version posted July 21, 2025. The copyright holder for this preprint (which was not certified by peer review) is the author/funder, who has granted bioRxiv a license to display the preprint in perpetuity. It is made available under aCC-BY 4.0 International license.

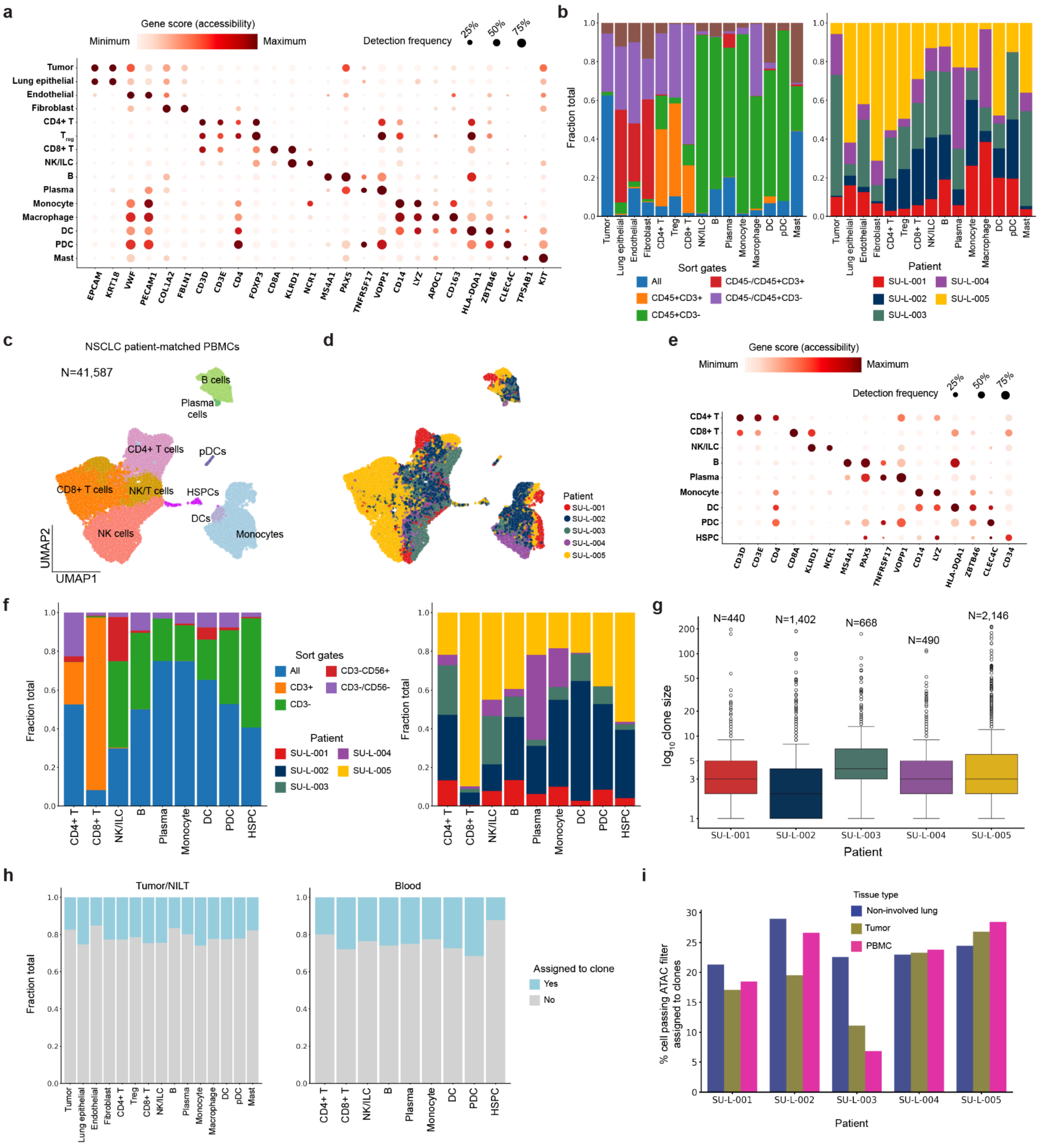

Supplemental Figure 2: NSCLC data summary and cell type annotation

(A) For cell types annotated in tumor and NILT samples, column-scaled gene accessibility scores and detection frequencies for the indicated genes.

(B) For tumor and NILT samples, bar plots indicating (left) relative proportions of markers used for sorting that were detected in each cell type (certain samples were not sorted, and select sorted samples were merged during single-cell capture, due to

bioRxiv preprint doi: https://doi.org/10.1101/2025.07.16.665245; this version posted July 21, 2025. The copyright holder for this preprint (which was not certified by peer review) is the author/funder, who has granted bioRxiv a license to display the preprint in perpetuity. It is made available under aCC-BY 4.0 International license.

sample-specific considerations to optimize single-cell yield) and (right) relative proportions of cells from each patient detected in each cell type.

(C) UMAP of 41,587 PBMCs from patients with lung tumors.

(D) UMAP of cells colored by patient identity.

(E) For cell types annotated in PBMC samples, column-scaled gene accessibility scores and detection frequencies for the indicated genes.

(F) For PBMC samples, bar plots indicating (left) relative proportions of markers used for sorting that were detected in each cell type (certain samples were not sorted, and select samples were additionally sorted using CD56 to enrich innate lymphocytes and myeloid cells) and (right) relative proportions of cells from each patient detected in each cell type.

(G) Clone size distributions for patients with NSCLC.

(H) Bar plots summarizing relative proportions of cells assigned to clones across cell types in tumor/NILT and blood. No significant cell-type bias was observed.

(I) Fraction of cells passing ATAC filters that are successfully assigned to clones.

bioRxiv preprint doi: https://doi.org/10.1101/2025.07.16.665245; this version posted July 21, 2025. The copyright holder for this preprint (which was not certified by peer review) is the author/funder, who has granted bioRxiv a license to display the preprint in perpetuity. It is made available under aCC-BY 4.0 International license.

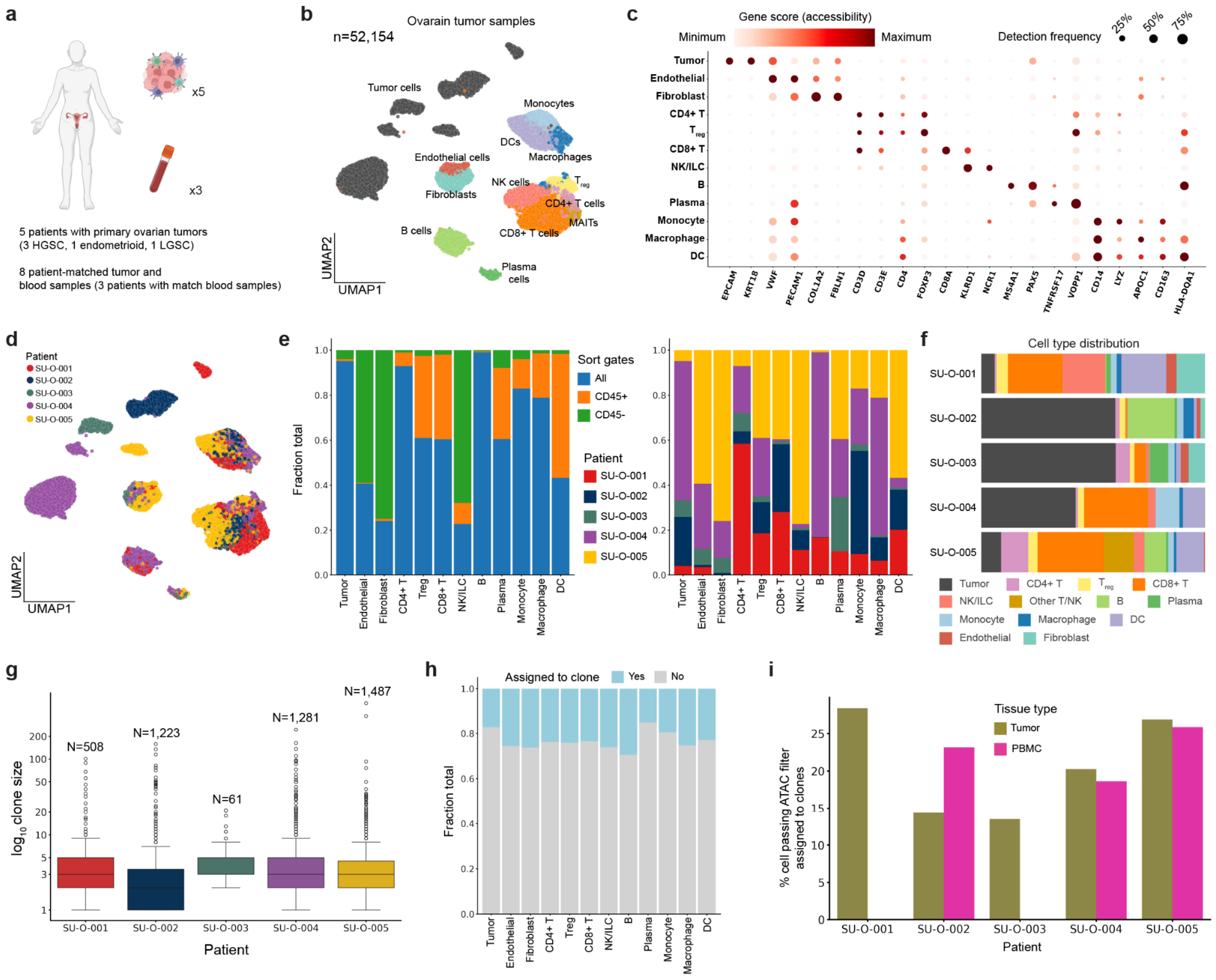

Supplemental Figure 3: Ovarian cancer data summary and cell type annotation

(A) Schematic summarizing patient and sample information for the ovarian tumor data.

(B) UMAP of 52,154 cells in ovarian tumors. Cell types denoted by color are inferred after iterative sub-clustering of each of the myeloid, lymphoid, and stromal compartments.

C) For cell types annotated in ovarian tumor samples, column-scaled gene accessibility scores and detection frequencies for the indicated genes.

(D) UMAP of cells colored by patient identity.

(E) For ovarian tumor samples, bar plots indicating (left) relative proportions of markers used for sorting that were detected in each cell type (certain samples were not sorted, and select sorted samples were merged during single-cell capture, due to sample-specific considerations to optimize single-cell yield) and (right) relative proportions of cells from each patient detected in each cell type.

bioRxiv preprint doi: https://doi.org/10.1101/2025.07.16.665245; this version posted July 21, 2025. The copyright holder for this preprint (which was not certified by peer review) is the author/funder, who has granted bioRxiv a license to display the preprint in perpetuity. It is made available under aCC-BY 4.0 International license.

(F) Normalized bar plot showing cell type composition for each patient.

(G) Clone size distributions for patients with ovarian cancer.

(H) Bar plots summarizing relative proportions of cells assigned to clones across cell types in ovarian and blood. No significant cell-type bias was observed.

(I) Fraction of cells passing ATAC filters that are successfully assigned to clones. Peripheral blood samples were obtained from three patients. Created with BioRender.com.

bioRxiv preprint doi: https://doi.org/10.1101/2025.07.16.665245; this version posted July 21, 2025. The copyright holder for this preprint (which was not certified by peer review) is the author/funder, who has granted bioRxiv a license to display the preprint in perpetuity. It is made available under aCC-BY 4.0 International license.

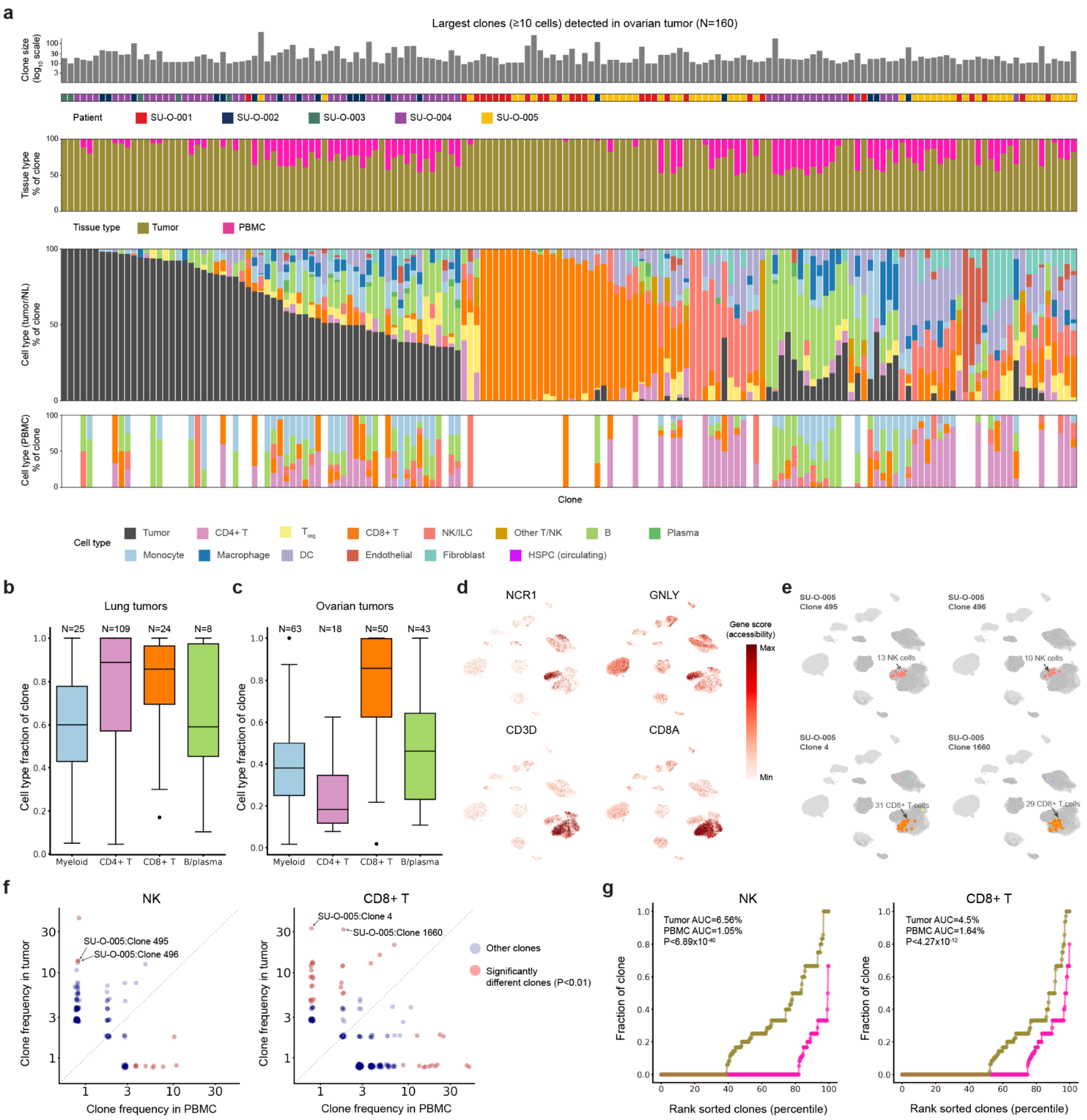

Supplemental Figure 4: Additional clonal landscape analyses

(A) All clones with at least 10 cells detected in ovarian tumor samples. Each column represents a unique clone.

(B and C) Clones with ≥5 cells of the indicated cell type are considered, and distribution of the indicated cell type's clone fraction is plotted. For myeloid cells, monocytes, macrophages, and DCs are grouped. For CD4^{+} T cells, T_{reg}s and other CD4^{+} T cells are grouped. N represents the number of clones considered for each indicated cell type. Analysis of lung tumor clones are displayed in (**B**) and ovarian tumor clones in (**C**).

bioRxiv preprint doi: https://doi.org/10.1101/2025.07.16.665245; this version posted July 21, 2025. The copyright holder for this preprint (which was not certified by peer review) is the author/funder, who has granted bioRxiv a license to display the preprint in perpetuity. It is made available under aCC-BY 4.0 International license.

(D) Expression of NK and CD8$^+$ T markers distinguish them on UMAP.

(E) Representative clones capturing clonal expansion events of NK cells (top row) and CD8$^+$ T cells (bottom row) in SU-O-005. For each clone, cells from the clone's donor are highlighted with shaded circles, and cells assigned to that clone are colored by their cell type.

(F) Scatterplots comparing clone frequencies of circulating cells with those infiltrating the tumor. The largest clones are locally expanded and minimally detected in the periphery. Significance is determined by Benjamini-Hochberg adjusted Fisher's exact test.

(G) Cumulative fraction of clone sizes for the indicated lymphoid cell types, split by tissue site. AUC corresponds to the overall clone size for the indicated cell type and tissue site. Kruskal-Wallis test.

bioRxiv preprint doi: https://doi.org/10.1101/2025.07.16.665245; this version posted July 21, 2025. The copyright holder for this preprint (which was not certified by peer review) is the author/funder, who has granted bioRxiv a license to display the preprint in perpetuity. It is made available under aCC-BY 4.0 International license.

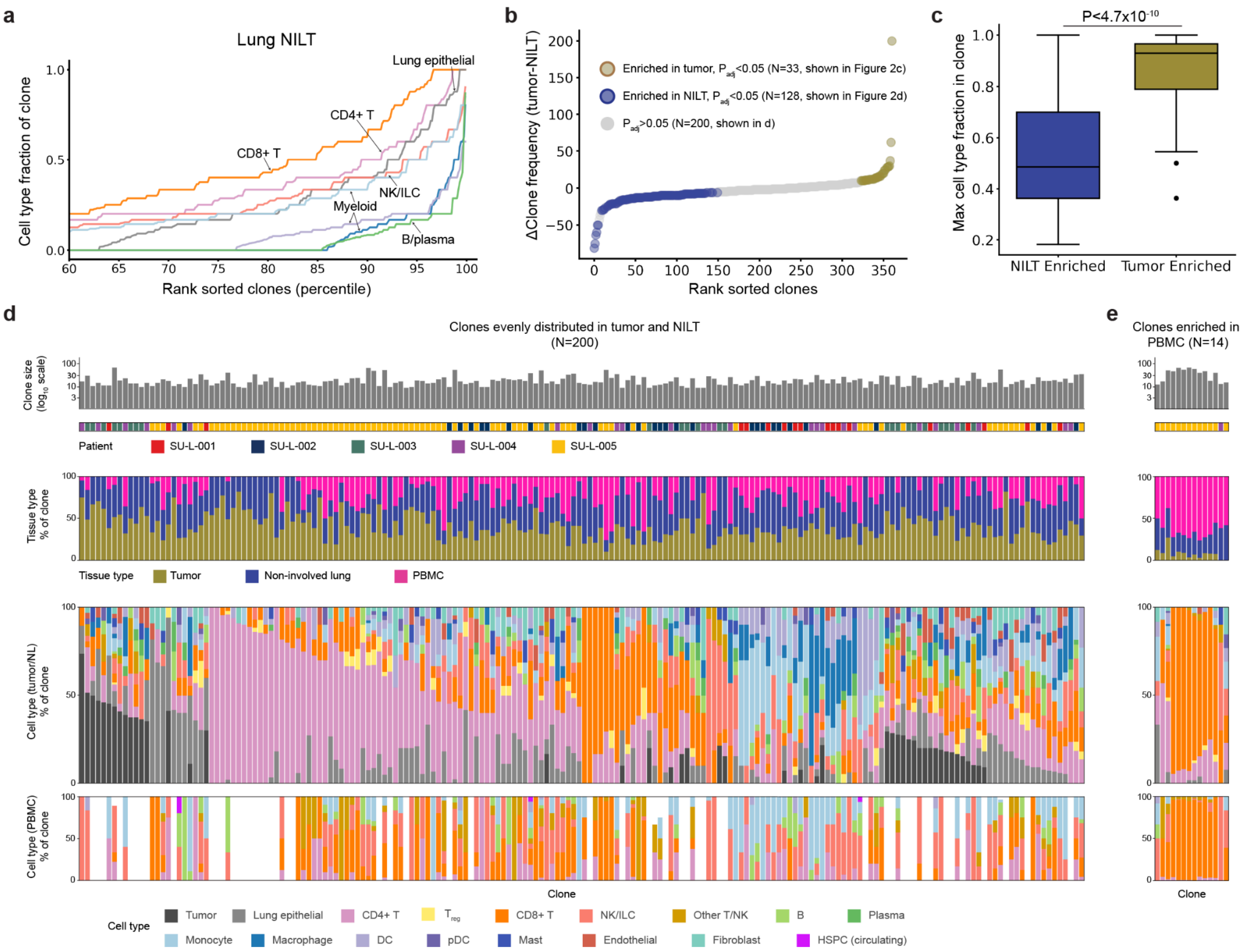

Supplemental Figure 5: Comparative clonal analysis across tissue sites in NSCLC

(A) Cumulative fractions of clones stratified by cell type for cells from NILT samples. Clones with ≥5 cells are considered for this analysis.

(B) Enrichment of clones in NILT or tumor. Significance is determined by Benjamini-Hochberg adjusted Fisher's exact test against overall tissue site distribution for clones with at least 10 cells in tumor and NILT.

(C) Comparison of dominant cell type fraction distribution between clones enriched in NILT and in tumor. Kruskal-Wallis test.

(D and E) Heatmaps showing the fraction of all cell pairs belonging to the same clone and consisting of two cell types within ovarian tumor (**E**) and PBMC (**F**). Pairs were restricted to cells from the same donor.

bioRxiv preprint doi: https://doi.org/10.1101/2025.07.16.665245; this version posted July 21, 2025. The copyright holder for this preprint (which was not certified by peer review) is the author/funder, who has granted bioRxiv a license to display the preprint in perpetuity. It is made available under aCC-BY 4.0 International license.

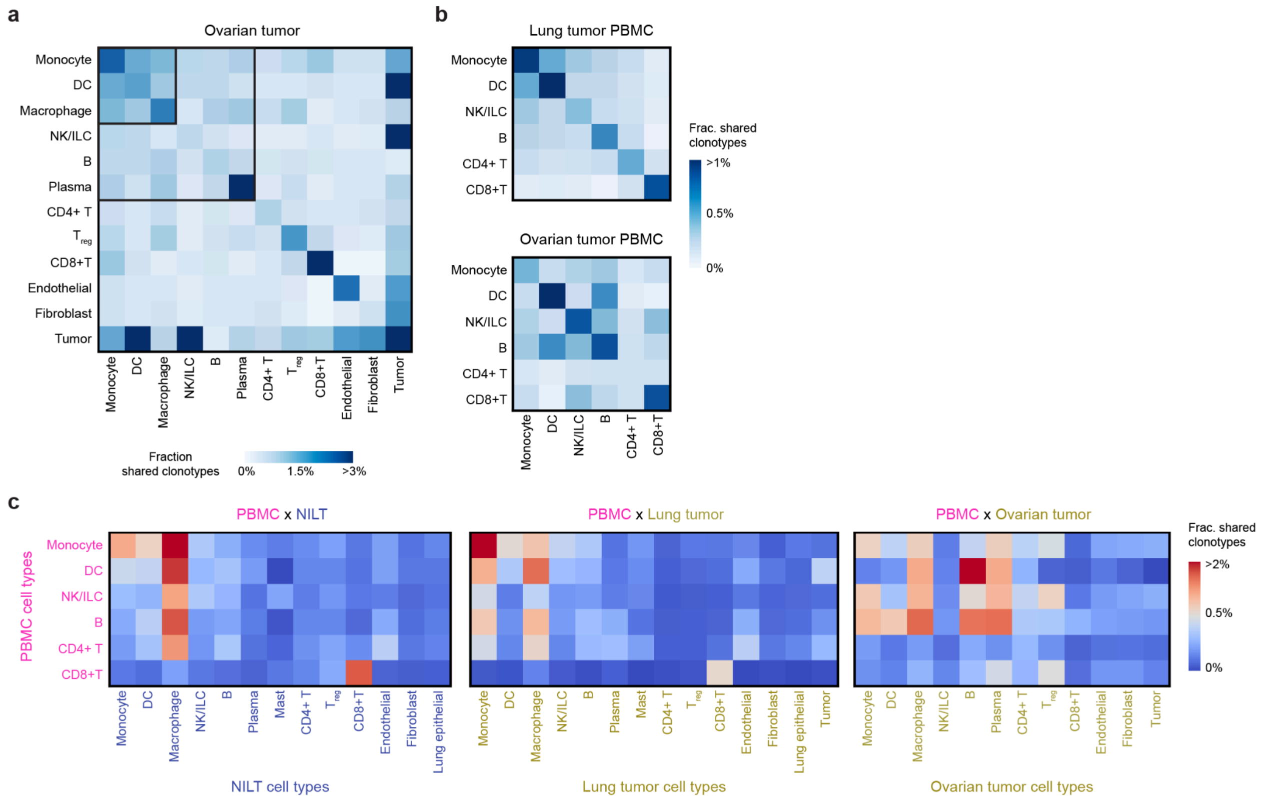

Supplemental Figure 6: Additional cross-tissue clonal analysis

(A and B) Heatmaps showing the fraction of all cell pairs belonging to the same clone and consisting of two cell types within ovarian tumor (**A**) and PBMC (**B**). Pairs were restricted to cells from the same donor.

(C) Heatmap showing the fraction of all cell pairs belonging to the same clone and consisting of a PBMC cell type and a solid tissue cell type.

bioRxiv preprint doi: https://doi.org/10.1101/2025.07.16.665245; this version posted July 21, 2025. The copyright holder for this preprint (which was not certified by peer review) is the author/funder, who has granted bioRxiv a license to display the preprint in perpetuity. It is made available under aCC-BY 4.0 International license.

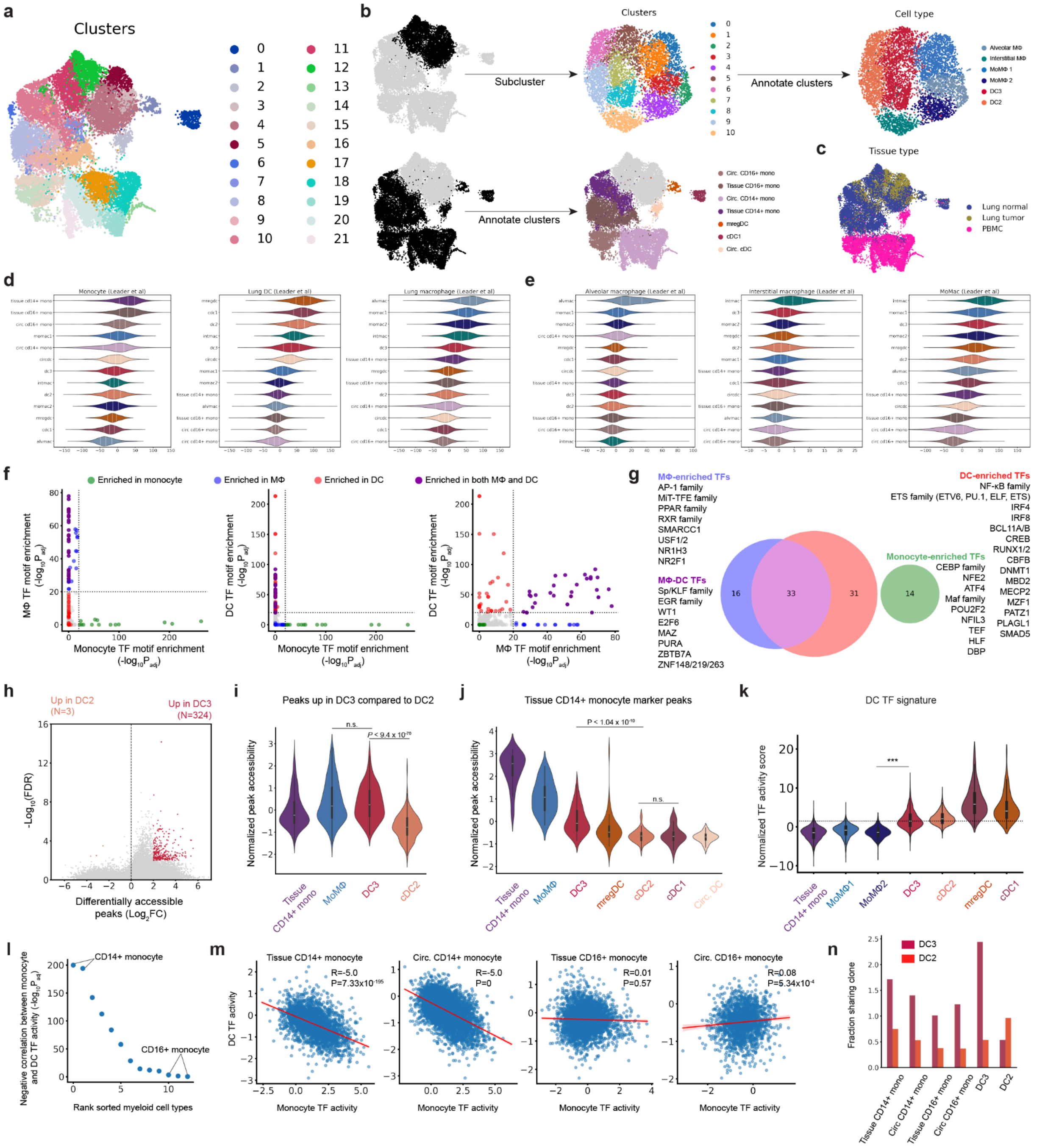

Supplemental Figure 7: NSCLC myeloid annotation and epigenetic analysis

(A) UMAP of myeloid cells from PBMC, lung tumor, and NILT samples of patients with NSCLC, colored by their original cluster assignments.

(B) Myeloid annotation scheme. Monocyte clusters separated clearly into CD14+ and CD16+ subsets, which were annotated without further subclustering. Macrophages and

bioRxiv preprint doi: https://doi.org/10.1101/2025.07.16.665245; this version posted July 21, 2025. The copyright holder for this preprint (which was not certified by peer review) is the author/funder, who has granted bioRxiv a license to display the preprint in perpetuity. It is made available under aCC-BY 4.0 International license.

DCs were subclustered to achieve higher granularity and annotated based on subclustering results.

(C) UMAP of cells colored by tissue sites.

(D and E) Gene scores of published signatures derived from single-cell RNA and proteomic data.

(F) TF motifs enriched in the marker peaks of three major MNP cell types. P-values are calculated from the Benjamini-Hochberg adjusted Wilcoxon signed-rank test.

(G) Summary of TFs whose motifs are enriched in broad MNP cell types. A $-\log_{10}P_{adj}>20$ cutoff was used.

(H) Differentially accessible genomic regions in DC3 vs DC2.

(I) Normalized sum accessibility of genomic regions significantly more accessible in DC3 compared to DC2 in indicated cell types.

(J) Normalized sum accessibility of genomic regions significantly more accessible in CD14+ monocytes compared to other myeloid subtypes.

(K) Average chromVAR motif deviation scores for DC TFs highlighted in **Figure 3C**. Kruskal-Wallis test.

(L) Statistical significance of monocyte-DC TF motif accessibility correlation in all myeloid subtypes. CD14+ monocytes in tissue and circulation display the most significant negative correlation between monocyte and DC TF motif accessibilities.

(M) Monocyte and DC TF activities are negatively correlated only in CD14+ monocytes but not in CD16+ monocytes.

(N) Monocytes share more clonotypes with DC3 than with DC2.

bioRxiv preprint doi: https://doi.org/10.1101/2025.07.16.665245; this version posted July 21, 2025. The copyright holder for this preprint (which was not certified by peer review) is the author/funder, who has granted bioRxiv a license to display the preprint in perpetuity. It is made available under aCC-BY 4.0 International license.

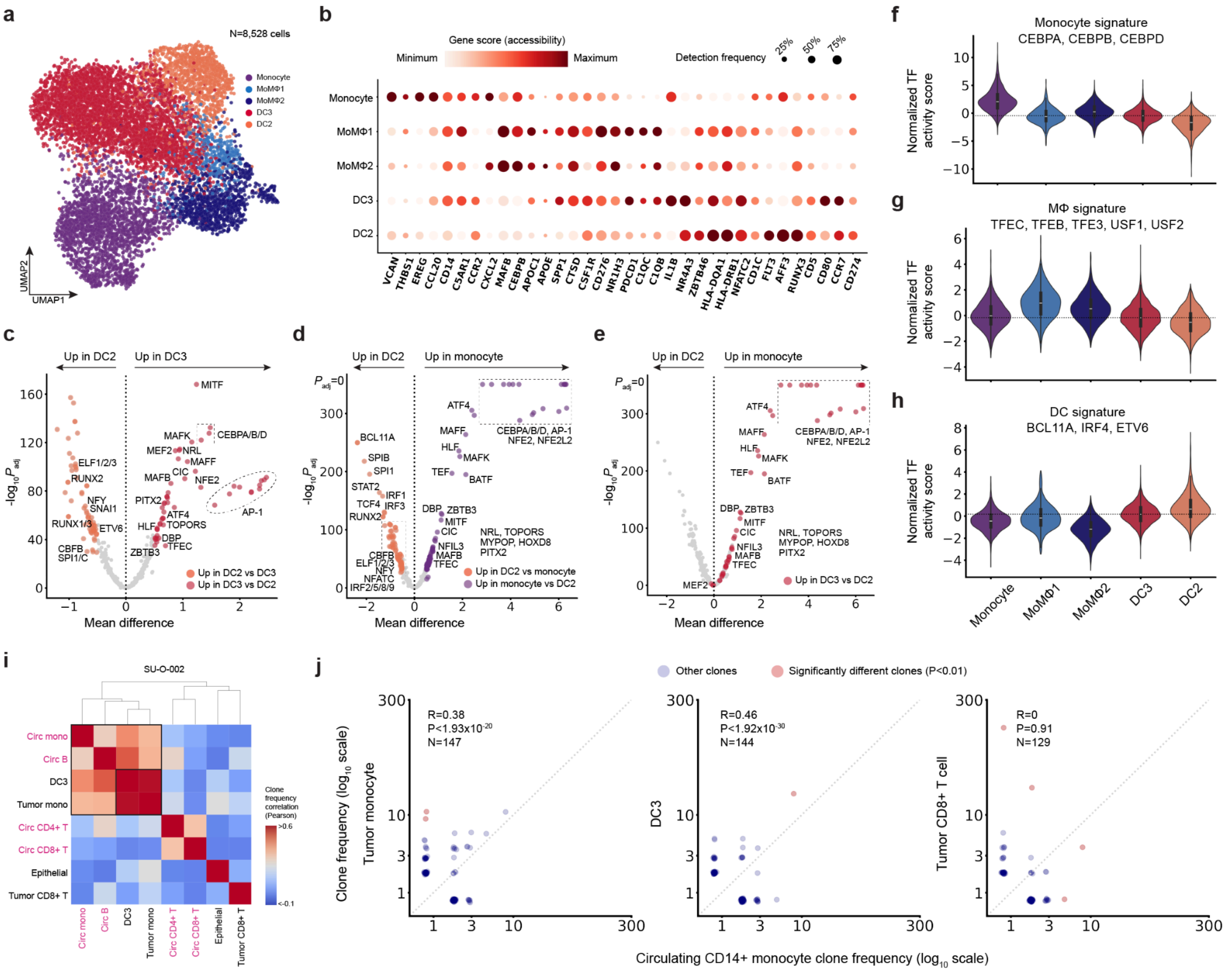

Supplemental Figure 8: Ovarian myeloid epigenetic analysis

(A) UMAP of myeloid cells from ovarian tumor samples.

(B) Column-scaled gene accessibility scores and detection frequencies for the indicated genes.

(C, D, and E) Differentially active TF motifs between DC2 and DC3 (**C**) and DC2 and CD14+ monocytes (**D** and **E**). DC3-up TF motifs from (**D**) are highlighted in red in (**E**). P-values are calculated using the Benjamini-Hochberg adjusted Kruskal-Wallis test.

(F, G, and H) Average chromVAR motif deviation scores for the indicated monocyte-, macrophage-, and DC-associated TFs.

(I) Cell type-cell type clone frequency correlation across clones (≥5 cells across all samples). Color denotes correlation value, computed using Pearson's ρ. Text labels of circulating PBMC cell types are colored pink.

bioRxiv preprint doi: https://doi.org/10.1101/2025.07.16.665245; this version posted July 21, 2025. The copyright holder for this preprint (which was not certified by peer review) is the author/funder, who has granted bioRxiv a license to display the preprint in perpetuity. It is made available under aCC-BY 4.0 International license.

(J) Scatterplots comparing clone frequencies of circulating CD14+ monocyte and tissue CD14+ monocyte (left), DC3 (middle), and tumor $CD8^{+}$ T cell (right). Significantly different clones with P<0.05 adjusted Fisher's exact test are highlighted red.

bioRxiv preprint doi: https://doi.org/10.1101/2025.07.16.665245; this version posted July 21, 2025. The copyright holder for this preprint (which was not certified by peer review) is the author/funder, who has granted bioRxiv a license to display the preprint in perpetuity. It is made available under aCC-BY 4.0 International license.

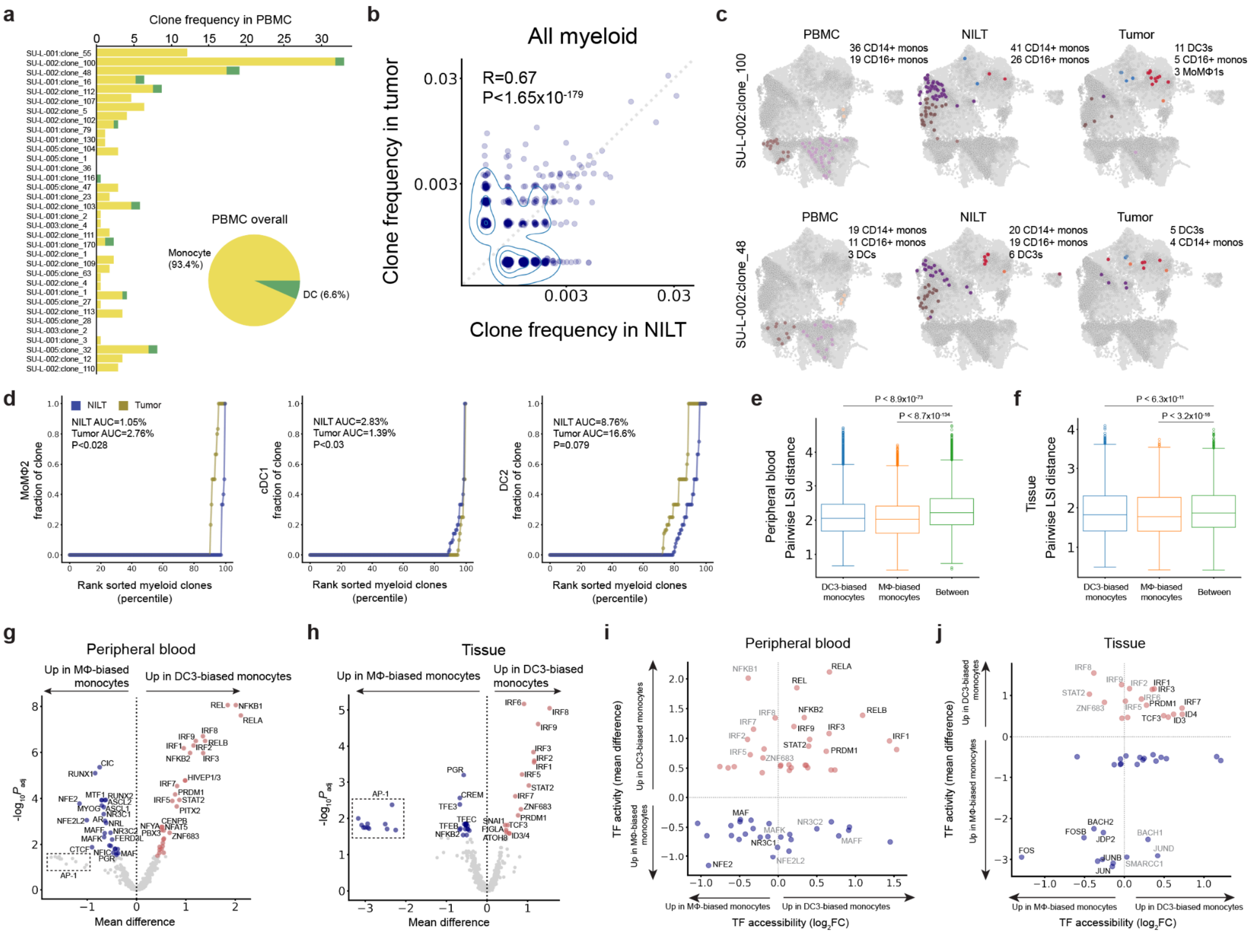

Supplemental Figure 9: Epigenetic comparison of DC- and macrophage-biased myeloid clones

(A) Monocyte and DC proportions of largest myeloid clones (≥10 cells) in PBMC samples of patients with NSCLC.

(B) Scatterplots comparing clone frequencies of myeloid cells in tumor with those in NILT. Contours visualize density.

(C) Representative clones capturing myeloid cell type distribution across tissue sites. For each clone, cells from the clone's donor are highlighted with shaded circles, and cells assigned to that clone are colored by their cell type.

(D) Cumulative fraction of clone sizes for the indicated myeloid cell types, split by tissue site. AUC corresponds to the overall clone size for the indicated cell type and tissue site. Kruskal-Wallis test.

(E and F) Epigenetic similarity as measured by distance in the LSI space within and between DC3-biased and macrophage-biased monocytes in circulation (**E**) and in tissue (**F**). Kruskal-Wallis test.

bioRxiv preprint doi: https://doi.org/10.1101/2025.07.16.665245; this version posted July 21, 2025. The copyright holder for this preprint (which was not certified by peer review) is the author/funder, who has granted bioRxiv a license to display the preprint in perpetuity. It is made available under aCC-BY 4.0 International license.

(G and H) Differentially active TF motifs between DC3-biased and macrophage-biased monocytes in peripheral blood (**G**) and in tissue (**H**). P-values are calculated using the Benjamini-Hochberg adjusted Kruskal-Wallis test.

(I and J) Joint comparison of TF gene body accessibility and inferred genome-wide TF activity to nominate TFs driving differential fate outcomes of monocytes in circulation (**I**) and in tissue (**J**). Select TFs that are both more accessible and motif accessibility are indicated in black, while TFs that only have increased motif accessibility are indicated in grey.
